# Neuroinflammation in Bipolar Depression

**DOI:** 10.3389/fpsyt.2020.00071

**Published:** 2020-02-26

**Authors:** Francesco Benedetti, Veronica Aggio, Maria Luisa Pratesi, Giacomo Greco, Roberto Furlan

**Affiliations:** ^1^ Psychiatry and Clinical Psychobiology Unit, Division of Neuroscience, IRCCS Ospedale San Raffaele Hospital, Milano, Italy; ^2^ University Vita-Salute San Raffaele, Milano, Italy; ^3^ PhD Program in Molecular Medicine, University Vita-Salute San Raffaele, Milan, Italy; ^4^ Clinical Neuroimmunology Unit, Institute of Experimental Neurology, IRCCS Ospedale San Raffaele, Milano, Italy

**Keywords:** bipolar disorder, neuroinflammation, cytokines, neuroimaging, neurotransmitters

## Abstract

Bipolar disorder (BD) is a leading cause of worldwide disability among mood disorders. Pathological mechanisms are still vastly unclear, and current treatments with conventional medications are often unsatisfactory in maintaining symptoms control and an adequate quality of life. Consequently, current research is focusing on shedding new light on disease pathogenesis, to improve therapeutic effectiveness. Recent evidence has suggested a prominent role of inflammation in mood disorders. Elevated levels of peripheral proinflammatory mediators have been reported in BD, as well as in other mood disorders, and people with systemic autoimmune diseases have an increased risk of developing BD. These immunological alterations are stable, and current medications are unable to alter peripheral concentrations even when clinical improvement is evident. These findings have also been replicated in the central nervous system (CNS) milieu, whereas genetic studies have shown that these immune alterations are not due to the disorder itself, being detectable before the illness onset. Moreover, these inflammatory modifications seem to be affected by and linked to other biomarkers of the disorder, such as alterations of white matter (WM) microstructure, metabolism, kynurenine pathway, and circadian rhythmicity. Finally, these immune variations seem to be useful as predictors of therapeutic responsiveness to medications, and in discriminating between clinically different outcomes. The objective of this review is to summarize available evidence on the connection between inflammation and BD, focusing on peripheral inflammatory markers and recent findings on their connection with other typical features of BD, to outline a general overview of the disorder. Moreover, it is meant to analyze the issues with data gathering and interpretation, given the partially contradictory and inconsistent nature of results.

## Introduction

At least 20% of the general population will experience a mood episode at some time in their lives or, even worse, develop a mood disorder. Within mood disorders, bipolar (BD) and major depressive disorder (MDD) are the most frequent and disabling ones. BD is a chronic recurrent mental disorder characterized by mood shifts, ranging from acute depression to mania and hypomania, and followed by a return to euthymia. BD affects around 2% of the general population, with a typical early onset before 30 years of age (1).On the other hand, MDD is characterized by a chronic recurrence of depressive episodes with a lifetime prevalence of about 12%. Patients generally reach clinical attention only years after the illness onset, with a delay of 6–10 years for a correct diagnosis. Because of the absence of specific biomarkers, the diagnosis is made simply with a clinical interview, thus leading to possible misdiagnosis, particularly for those BD patients that experience a clear hypomanic or manic episode only after a few depressive ones. Clinical and physiological manifestations of BD are complex, heterogeneous, and severe mood changes are only the most evident sign, as it also involves neurovegetative, inflammatory, molecular, metabolic, and psychomotor alterations. Moreover, the efficacy of current medications, mainly antidepressants and mood stabilizers, is modest for both disorders and has a delayed effect. Although several risk factors have been identified, mainly genetic and environmental, the pathophysiology of these disorders is largely unknown. One of the most reported biomarkers of the disorder is a widespread alteration of white and grey matter microstructure (2–4), together with an atrophy of the hippocampus (5), but their biological underpinning still remains unclear. Following this line of reasoning, it is clear why the psychiatry research field is looking for new biomarkers that can be helpful to improve the etiology, diagnosis, and treatment understanding of BD.

## The Immune System in BD

Evidence for possible involvement of the immune system, and in a broader sense, inflammation, in mood disorders, has been steadily accumulating in recent years. The rationale behind this relation originally arises from the observation of the so-called “sickness behavior,” the complex mixture of behavioral changes observed in inflammatory states, which includes mood changes such as anxiety, depression, failure to concentrate, lethargy, and more. Proinflammatory cytokines such as tumor necrosis factor-α (TNF-α), interleukin-6 (IL-6), and interleukin-1β (IL-1β) are held responsible for these changes (6). It has also been established that these cytokines can induce MDD in physically ill patients without a previous mental illness (7). Moreover, a great percentage of patients with mood disorders show comorbidities with autoimmune pathologies, such as hypothyroidism, rheumatoid arthritis, diabetes mellitus, hepatitis, and Crohn's disease (8, 9). Patients who experience systemic autoimmune diseases also present an increased risk of developing BD (10). Consequently, these findings prompted further research into the potential role of cytokines and immunity markers on affective disorders.

Even if the data are not always convergent, the majority of the studies reported increased peripheral cytokines levels in both MDD and BD. The greater limit of these studies is the use of peripheral blood for the analysis of immune markers, thus leading to many questions about whether the same alterations could be observed in the central nervous system (CNS). This is a core point, considering the fundamental importance of CSN-specific T-cells' role in shaping brain function (11, 12). Some elegant studies have analyzed the CNS inflammatory status through microglia activation. Microglia are probably the most important innate immune cells in the brain, where they regulate cellular inflammatory response and represent the primary source of proinflammatory cytokines. Haarman and colleagues (13) firstly reported a neuroinflammatory condition *in vivo* in BD using positron emission tomography (PET). *In vivo* microglia characterization showed significantly increased activation in the hippocampus of BD patients compared to healthy controls (HC). Furthermore, in a subsequent study, the same group reported a direct association between microglia activation and neuronal damage in BD, thus suggesting a possible harmful effect of this neuroinflammatory condition (14). Previously reported findings of inflammatory markers in the periphery now seem to be supported by microglia studies, thus contributing to create more evidence supporting the neuroinflammatory theories on mood disorders.

One of the most recent theories about immune alterations in mood disorder is the one formulated from the reported findings of Grosse, Drexhage, and colleagues. This theory postulates that MDD and BD share a partial deficit at different age periods; MDD patients have an early mild T cell defect, characterized by a reduced maturation of Th2 and Th17 cells, that becomes more prominent with increasing age, also including a reduced maturation of T regulatory cells and an increased immune activation (15). On the other hand, BD could be characterized by an early T cell defect similar to those of MDD aged patients, which will become partially restored with increasing age (16). More evidence supporting this hypothesis comes from genetic studies: aberrant expression of mRNA inflammatory genes in BD and related offspring has been reported. This “genetic signature” is present before illness onset, thus underling that an inflammatory condition—due to a different genetic expression—is not the consequence of the disorder itself, but more likely one of the contributing factors (17). In a subsequent study on BD twins, the same group found that the occurrence of the previously reported gene-expression signature was principally due to shared environmental factors, demonstrating that BD itself cannot be considered as a causative factor for monocyte activation (18). The involvement of immune dysfunctions as a consequence of different genetic expressions would therefore seem a plausible etiological factor implicated in BD onset and development. In the next subheadings we will present and discuss the findings on peripheral markers in BD, and we will then proceed to link these findings to other core features of BD, such as brain structural alterations, neurotransmitter production and release, circadian alterations, and sleep disturbances.

## Peripheral Immune System Alterations in BD

### Innate and Adaptive Immunity Inflammatory Cytokines

During the last few years, increasing evidence for the involvement of the immune system in mood disorders has been accumulating, with many studies showing significant changes to cytokines levels. Cytokines are small molecules secreted by the immune system in response to a variety of stimuli, which are capable of affecting the behavior of other cells. More in detail, cytokines are capable of control and promote cell and tissue growth, development, migration, differentiation, and death. In BD, significantly elevated levels of IL-1ß were detected in the serum of BD patients during manic episodes compared with HC, whereas those experiencing depressive episodes did not show variations (19). Data on IL-6 are among the most consistent in the whole literature on BD. IL6 is a pleiotropic cytokine which is known to enhance clonal expansion and activation of T cells, B cells differentiation, and to manage acute- phase response (20). A vast majority of immune system cells can produce IL-6 when driven by Toll-like receptors (TLR), prostaglandins, adipokines, stress responses, and other cytokines. IL-6 levels were found to be higher in BD patients compared with HC. Moreover, they seemed to considerably decrease after 6 weeks of treatment (21). The same study demonstrated an increase in TNF-α as well, although no significant reduction was noticed after treatment. These data were also confirmed by a recent meta-analysis (22). Evidence supporting the role of an inflammatory milieu in the pathogenesis of BD is growing. This could perhaps play a part in the increase in mortality and clinical comorbidities observed in BD patients.

Among all factors involved, TNF-α holds a key position. TNF-α has a prominent role in the regulation and preservation of immune system mechanisms, inflammation, and host defense. It has also been associated with chronic inflammation, autoimmunity, the development of cancer and the metastatic process (23). Numerous studies reported elevated TNF-α levels in BD patients, as opposed to HC, during both manic and depressive episodes(19, 22, 24).Nonetheless, some results suggest that this marker may not be significant at all (25, 26). Such inconsistencies may be related to heterogeneity in the samples, due to discrepancies in symptoms, disease duration, and treatment. Some studies explored the significance of TNF receptors (TNFR1 and TNFR2), which appear to be more stable and could, therefore, represent more dependable markers than the cytokine itself. Moreover, these receptors are likely to be involved in BD, since various studies described their alteration in other conditions, such as MDD, schizophrenia, and Parkinson's disease. In particular, the soluble isoform of TNFR1 (sTNFR1) showed a strong association with BD patients in any phase of the disease, as compared to HC, although the same did not apply to sTNFR2 (27).

A marker which gained quite some attention in this field was CRP. A recent meta-analysis (28) found that CRP concentrations are higher in BD patients, substantially during a manic episode, moderately throughout depressive episodes or euthymia. These data were partially corroborated by another meta-analysis (29), which confirmed elevated levels of CRP in manic and euthymic, but not depressed BD patients. Further findings regarded different increases in CRP concentrations in distinct mood shifts. Results showed that notwithstanding evidence of higher levels in all three phases when compared with healthy subjects, CRP rises more in the manic stage compared to depression and euthymia in BD. Furthermore, CRP concentrations seemed to be higher in patients who did not undergo any treatment. Despite great evidence for the involvement of CRP in BD, the extension of its elevation was not associated with symptoms severity or disease duration. Besides, CRP levels decreased when achieving euthymia after a manic episode, without reaching control groups concentrations. The same study also demonstrated that high concentrations of CRP raise the risk of developing BD, but do not cause neuroprogression in BD patients (30). However, other studies reported no significant differences between HC and BD patients, although trends could be observed for a reduction in CRP levels for manic and depressive periods compared with euthymia (31).

Among the adaptive immune cytokines, the most relevant ones analyzed include IL-2 and INF-γ. Results from literature were somewhat inconsistent, as a few studies reported no differences between BD patients and HC (21, 32), whereas others noted a significant reduction of IL-2 levels in BD patients (19). Some studies found INF-γ to be reduced (33) andothers reported it as increased in BD patients anda reduction in its levels was evident after a few weeks of pharmacological treatments (34).

The accumulation of a vast amount of evidence in favor of a proinflammatory activation in BD pathogenesis has led to the belief that antiinflammatory cytokines in affected subjects would be reduced or unchanged. Many studies reported no difference between IL-10 levels in BD patients and HC (24, 35). Nonetheless, there were a few contradictory reports. Other works found elevated antiinflammatory cytokines levels, similarly to findings for major depressive disease, as a compensatory response to proinflammatory mobilization (22). A meta-analysis, which included over 1,600 among healthy subjects and BD patients in various phases of the disease, demonstrated significantly higher levels of IL-4 in BD patients compared to controls (22).

It should also be considered that the immune system is deeply connected to the circadian system. The link between these two entities takes place on multiple levels. On one hand, the immune system features daily cycles, which seem to be vital for the retention of a functional apparatus. On the other hand, immune responses cause changes in circadian rhythms, disrupting their stability. Many immune cell types show daily fluctuations in human and rodents' blood. Among those are T and B cells, monocytes, macrophages, and NK cells. The same is true for various cytokines, such as IL-1ß, IL-6, INF-γ, and TNF-α. Many of the studies on this topic were performed on regular day-night and sleep-wake conditions, therefore rendering it impossible to understand if differences in concentration were due to the endogenous circadian system or cyclical environmental signals.

### Chemokines

Studies exploring the association between BD and chemotactic cytokines are sparse. One of the most relevant had enrolled 70 BD patients and 50 healthy subjects (36). BD patients presented higher levels of CCL11, CCL24, and CXCL10, and reduced levels of CXCL8 compared to controls. No significant differences were noted between manic and euthymic patients. Higher CXCL10 have also been reported in a separate study (37). CXCL10 promotes activated Th1 cells mototaxis, and Th1 cells hyperactivity has already been demonstrated in BD patients. On the other hand, CCL11 and CCL24 activity is suggestive of Th2 cells hyperactivity, which is known to be associated with BD.

Lastly, CXCL8 is a chemokine produced by monocytes, macrophages, and endothelial cells. Previous research has highlighted contradictory data concerning BD, with some of the works finding an increase in its plasmatic levels for depressive and manic states, while others did not report alterations in euthymic patients.

## Inflammatory Status Across Mood States: A Roundup

Even if quite the majority of the studies seems to point out a peripheral inflammatory markers alteration in BD (38), data available is heavily subject to interpretation, and we will now produce a brief roundup of cytokines movement throughout different mood states, extrapolated from what we already discussed.

Among the most prominent cytokines in human biology are tumor necrosis factor-α (TNF-α), interleukin-6 (IL-6), and interleukin-1β (IL-1β). These are heavily implicated in many physiological processes, and it is of huge interest to seek their potential role in BD mood swings.

As far as IL-1ß goes, a study showed that BD patients during manic episodes produce higher levels of the cytokine, whereas those experiencing depressive episodes did not show variations (19).

On the other hand, IL-6 levels were found to be elevated in BD patients, across all mood states and without significant correlation to the polarity of mood states. A crucial finding is that they were notably reduced after 6 weeks of treatment (21), something which could hold a very significant value in assessing the efficacy of pharmacological treatment in these patients, and therefore adjusting therapeutic approaches. TNF-α was also shown to be increased in BD patients in the same study, even though its levels did not significantly decrease after treatment.

Still, TNF-α was assessed in many different studies, with contrasting results. A few studies asserted that BD patients generally show higher levels of TNF-α than HC, with no significant difference between manic and depressive episodes (19, 22, 24). At the same time, some studies concluded that no such difference existed (25, 26). Such inconsistencies highlight the need for more dependable biomarkers, which could prove to be more stable and consistent across patients and mood states. TNF receptors (TNFR1 and TNFR2), are potential candidates for the role, and indeed the soluble isoform of TNFR1 (sTNFR1) demonstrated strong correlations with BD patients in any phase of the disease, as compared to HC, although the same did not apply to sTNFR2 (27). Further studies are surely needed to better assess the potential of this interesting marker in such patients.

Another very promising marker, implicated to a great extent in a number of different milieus, is CRP. Data in the field are again inconsistent and generally come from large meta-analysis, which all show higher CRP concentrations in BD patients compared with HC, but are not on the same page as it regards levels through various mood states in BD.

A few studies also looked at adaptive immunity cytokines, notably IL-2 and INF-γ, which would implicate a more complex pathological mechanism. Indeed, results showed an even higher degree of variability, although some showed promise in highlighting possible markers of response to treatment, particularly one which reported a reduction in INF-γ levels after a few weeks of pharmacological treatment. For summing up purposes, we hereby provide a table with all major findings on cytokine levels in studies included in this review (**Figure S1**).

The overall direction from the mentioned studies is in favor of a proinflammatory activation in BD pathogenesis, but these results taken together push the need to find a better way to standardize methods such as patients' selection, samples collection timing, storage and analysis, among others. Efforts shall be put towards standardizing all studies which aim to explore such correlations among peripheral cytokines, so as to compare results more objectively, and hopefully find more consistency when putting data together.

## More Than Autoimmunity: Other Proposed Factors Contributing to BD Etiology

Before the interest in immune alterations as a possible contributing factor to BD pathology, several decades of research have enlightened different systemic and local alterations as biomarkers of the disorder. The most-reported ones relate to brain structure, neurotransmitter release and neurocircuit functioning, circadian rhythmicity, tryptophan degradation, metabolism, and cognitive modification. These markers are both interconnected and linked to the immune system modification found in BD.

## Brain Structural Alterations

White matter (WM) microstructural alterations have been extensively associated with BD and have been proposed as a possible biomarker of the disorder. Neuroimaging studies on diffusion tensor imaging (DTI) consistently reported a widespread pattern of increased diffusivity perpendicular to the WM tracts' main diffusion trajectory (radial diffusivity, RD), and in the molecular diffusion rate measured by mean diffusivity (MD) (2, 39–41). These patterns of alterations are usually accompanied by a reduced diffusivity along the main WM fiber axis, or axial diffusivity (AD), and a lower coherent directionality, measured by fractional anisotropy (FA). WM alterations also concern a volumetric reduction, as described in voxel-based morphometry studies (42), and increased WM hyperintensities (43, 44). These WM modifications have been linked to possible alterations in oligodendrocytes, the axons' myelinating cells in the CNS (45). In the brain of individuals with BD, postmortem studies show a significant reduction in oligodendroglial cells density (46–48), and oligodendrocyte-specific mRNA markers were found to be downregulated (49), thus possibly impairing the homeostatic maintenance of myelinated axons and re-myelination processes (50).

Moreover, stressed oligodendrocytes can also lead to microglia activation through the release of different signaling chemokines and cytokines (51). Microglia activation, as previously reported, has been found in postmortem and *in vivo* studies of BD patients. In physiological condition, microglia is one the major glial cell populations and is present in high density in all adult brain regions. In a “resting” condition, microglial cells are morphologically characterized by a small cell soma with several elongated processes, which scan the surrounding environment. If these cells meet harmful stimuli or pathogens, they retract the processes and become more mobile, gaining effector abilities. This microglia condition, called M1 state, has prominent proinflammatory features, with the release of cytokines, chemokines, and reactive oxygen species (ROS). On the other hand, microglial cells can move into a M2 condition to downregulate, repair, or protect the brain from inflammation (52, 53). If this physiological activation into a proinflammatory state becomes chronic, it can drive neuronal damage and synaptic alterations, thus leading to neurodegenerative diseases (54).

We investigated the hypothesis that WM microstructure alterations, that have repeatedly been found in BD, could be linked also to a neuroinflammatory condition. In their first research, higher peripheral levels of inflammatory cytokines, such as TNF-α, IFN-γ, IL-8, IL-10, IGFBP2, and PDGF-BB were positively associated with RD and MD, and negatively with FA. These initial findings seem to suggest that a higher concentration of cytokines can be negatively linked to the integrity of myelin sheaths (55). To investigate inflammation not only *via* a multiplex immunoassay but also through immune characterization, the subsequent study investigated the levels of circulating T cells characterized *via* flow cytometry analyses. Peripheral T-helper 17 cells were positively associated with FA, whereas T regulatory cells (FoxP3 positive) were positively associated with RD. These findings were found also in a control sample of healthy volunteers. This balance between regulatory and helper-17 T cells seems to be fundamental not only for the immune homeostasis but might also regulate WM microstructure (56).

Another recent study reported that reduced frequencies of circulating CD8+ effector T cells related to decreased FA and increased RD, independently from BD illness phase (57). Altogether, these studies seem to support the idea that WM alterations found in BD can be related to neuroimmunological alterations. Even if all of these studies have been conducted on peripheral quantification and/or characterization of inflammatory markers, we can hypothesize that a similar condition can also be present in the CNS. Peripheral cytokines can enter the brain by volume diffusion, or *via* active cytokine transporters at the blood-brain barrier (BBB) (7). Further research will be instrumental in clarifying this theory.

Moreover, Patel and colleagues have suggested that the BBB of BD patients can be impaired transiently or persistently, thus leading to a facilitate passage of proinflammatory molecules from the periphery and decreased CNS protection. This BBB disruption can be linked to the neuroinflammatory alterations, microglial activation, and oligodendrocytes dysfunction previously described in BD (58). The demonstrated presence of a relation between one the most recognized biomarker of the disorder, WM pathology, and an inflammatory alteration, seems to underlie that the immunological investigation of BD can lead to a novel and, maybe, completely different understanding and conceptualization of its etiology, suggesting a new field of therapeutic investigation.

Moreover, cortical grey matter (GM) volume reduction in schizophrenia has been associated with higher expression of IL-6, IL-1β, IL-8, and SERPINA3 mRNAs in the prefrontal cortex of postmortem brains (59). Unipolar and bipolar depressed patients with a family history of mood disorders showed a 48% and 39% reduction in subgenual PFC volume, respectively (60), because of a reduction of glial cell number and density and of a reduction of synapses with a preserved number, and increased density, of neurons (61–63). GM volumes in the orbitofrontal cortex of patients with BD and their healthy siblings are smaller than in unrelated HC (64), and are even smaller when BD is associated with severe symptoms such as suicide (65). It could be hypothesized that cytokines play a role in GM reductions in BD, too. In the few available studies, proinflammatory cytokines were associated with GM volumes in the orbitofrontal cortex, lingual gyrus, inferior frontal cortex, middle frontal cortex (66), but with reduced effects in BD compared with schizophrenia (67). We studied stem cell factor (SCF), which is a hematopoietic growth factor and a neurotrophic factor, involved in neuron-neuron and neuron-(micro)glia interactions, fostering neuronal growth and an antiinflammatory milieu; and correlated SCF levels with antidepressant response and with functional and structural MRI measures in cortical areas that are involved in the cognitive generation and control of affect, thus suggesting that factors affecting the inflammatory state in the brain do influence cortical structure and function in BD illness episodes (68).

## Neurotransmitters: The Kynurenine Pathway of Tryptophan Degradation

One of the first hypotheses concerning mood disorders etiology was related to neurotransmitters alterations: psychopharmaceutical development has highlighted an important role of different neurotransmitter systems into the functional and clinical aspects of mood disorders. However, although existing literature tends to emphasize specificity in drugs' mechanisms of actions, many effective treatments target multiple mechanisms and a multitarget approach to treatment could be better suited for a multifactorial illness such as MDD (69).

MDD has been initially linked to monoaminergic alterations, such as serotonin, dopamine, and norepinephrine, and the gold standard drugs specifically target monoamine neurotransmitters to augment their activity *via* reuptake inhibition mechanisms (e.g., selective serotonin reuptake inhibitors or SSRIs) (70). This monoaminergic theory has been partially rejected only decades after, due to increasing evidence, such as the absence of depressive symptoms in healthy subjects with experimentally induced monoamine depletion (71), moving towards the idea that monoaminergic alterations only partially contribute to explain MDD clinical features. On the other hand, switches between depression and manic episodes in BD patients have originally been linked to changes in dopaminergic systems functioning. Depressive features were believed to be underlined by a hypodopaminergic condition, whereas the manic phase would have been due to a hyperdopaminergic condition (72). Dopamine antagonists and partial agonists are widely used in the pharmacological treatment of BD, the use of which range from depressive episodes to acute manic phases. These neurotransmitters systems, which are altered in BD, are also regulated by immunological modification. Under physiological conditions, cytokines are fundamental players in synaptic plasticity, neurogenesis, learning, and memory-linked mechanisms (73). Some immunological alterations, like those reported in BD, can also influence the neurotransmitters systems involved in BD.

Two of the previously described cytokines, TNF-α and IFN-γ, play a major role in influencing one fundamental biological pathway in the brain: the kynurenine pathway of tryptophan degradation. Alterations in the kynurenine pathway, that begins with tryptophan degradation, have been hypothesized in depression models; this pathway is the starting point for serotonin and melatonin biosynthesis, both quantitatively altered in BD (74–77).

Serotonin synthesis rate is determined by the bioavailability of tryptophan. Tryptophan degradation is operated by indoleamine 2,3 dioxygenase (IDO) or tryptophan 2,3 dioxygenase (TDO). The pathway regulated by IDO is particularly important for immune homeostasis and regulation, especially during infection and autoimmunity (78). In an inflammatory condition, such as those triggered by the presence of TNF-α and IFN-γ, peripheral tryptophan is converted by IDO into kynurenine. Once transported in the brain, kynurenine degradation leads to the formation of either 3- hydroxykynurenine (3-HK) and quinolinic acid (QUIN) or kynurenic acid (KA) (7). KA show a neuroprotective effect, competitively antagonizing NMDA glutamate receptors, where 3-HK and the derived QUIN seems to exert neurotoxic effects (79). Following IFN-γ activation in the IDO pathway, the kynurenine system exerts an antiinflammatory effect *via* KA releasing, to modulate immune response (78). An imbalance of the kynurenine pathway of tryptophan degradation can lead to an ineffective inflammatory counterbalance activity, and neurotoxicity.

Several studies have shown an involvement of this kynurenine pathway of tryptophan degradation in BD and MDD, which seems to be shifted towards its neurotoxic branch (80, 81). In a cutting-edge study, Myint and colleagues report that plasmatic levels of kynurenine out of tryptophan, defined as tryptophan breakdown index, was increased in BD, together with a reduction of KA concentration, thus lowering its neuroprotective effect (81). More recently, Birner and colleagues report similar data of decreased KA levels in a BD sample compared to healthy volunteers (82). Finally, kynurenine breakdown has been related also to WM microstructure, where BD show reduced concentrations of KA and 5-HIAA, a measure of serotonin levels, that are positively associated with DTI measures of WM integrity (83).

Moreover, ventral, striatal-ventrolateral, and orbitofrontal cortical reward processing circuitry have been proposed to define an endophenotype specific of BD (84), and consistent evidence associates inflammation with decreased dopamine synthesis, packaging, and release, leading to decreased dopamine and dopamine metabolites in cerebrospinal fluid, and decreased availability of striatal dopamine, which results—at the behavioral level—in depressive symptoms related to motivation and motor activity (85).

## Circadian Rhythms and Sleep Alterations

Circadian rhythm alterations and sleep disruption have been closely associated with BD pathophysiology (86–88). The fundamental circadian pacemaker is located in the suprachiasmatic nucleus of the hypothalamus, based on multiple transcriptional/translational feedback loops, and it hierarchically controls the peripheral clocks (89). Immune responses cause changes in circadian rhythms, disrupting their stability. Many immune cells types show daily fluctuations in human and rodents' blood. On the other hand, both circadian rhythms and sleep plays a regulatory role on immune system: sleep is involved into the maintenance of Th1 and Th2 homeostasis, the activity of effector and regulatory t cells, and natural killer (NK) cells (90). Sleep seems also to be involved in the transcription regulation of pro and antiinflammatory factors (91).

The influence of sleep alteration of immune functions has been demonstrated also in animal models: sleep deprivation promotes an inflammatory condition *via* microglia and astrocytes activation in rats (92), thus leading to increased proinflammatory cytokines release. Another study reports that in mice with a predominant proinflammatory response, there is an increased BBB permeability and neuroinflammatory markers expression (93). It is possible to surmise that the chronic inflammatory condition that seems to characterize a subpopulation of BD patients can, therefore, also be related to sleep and circadian disturbances. Studies are lacking on the topic, but in a very small sample, major depression was associated with significant diurnal elevations in plasma interleukin-6 levels, a shift of its circadian rhythm, and loss of physiological complexity in its secretion, resulting in the presence or absence of significant differences in respect to healthy subjects at different circadian time points (94).

## Adipose Tissue Involvement

BD patients usually present with chronic clinic comorbidities, which cause increased mortality from cardiovascular, respiratory, and endocrine disease. Although currently available treatments (mood stabilizers and antipsychotics) are associated with weight gain, some studies suggest that somatic types with significant abdominal fat carry a higher risk of developing BD (95). Recent findings suggest an association between BD and weight gain, independent from pharmacological treatment. Possible mechanisms supporting the relation between BD and obesity include endocrine dysregulation, behavioral patterns (physical inactivity and excessive food intake), and a proinflammatory state connected to obesity (96).

Adipokines, such as adiponectin, resistin, and leptin, are cytokines released from adipose tissue, and play a key role in energetic homeostasis, insulin sensitivity, and immune response (97). Recent work has shown that BD patients present with an increase in plasmatic levels of adiponectin and leptin as opposed to HC. Moreover, antipsychotic drugs did not seem to affect concentrations of these molecules (95, 98), but contrasting findings have been reported (25). About leptin, many heterogeneous results have been described. Some have reported a diminution in leptin levels for BD patients compared with controls (99, 100), whereas others have noted similar levels between the two groups (101). On a side note, a few works suggested that physical exercise in mood disorders patients could lower proinflammatory cytokines levels (102, 103). This effect might also be explained by physical exercise enhancing cortisol release, which in turn inhibits T cells cytokines production.

Finally, an association between body mass index (BMI) and WM microstructure has been found in BD: higher BMI was negatively associated with FA and positively with MD. Moreover, serum levels of triglycerides, glucose, and cholesterol inversely correlates with FA and directly with AD, RD, and MD (104). Altogether, these last findings seem to suggest that an increased BMI could be associated with reduced structural integrity, bundle coherence, and axonal branching, and demyelination in general.

Moreover, in the obese adipose tissue, M1-polarized macrophages can secrete inflammatory cytokines (105), which are thought to play a role in metabolic dysregulation and insulin resistance (105, 106), all contributing to hamper antidepressant response (107). In agreement with this hypothesis, we showed that BMI can indirectly hamper antidepressant response by increasing the levels of proinflammatory cytokines (108). Given that an altered WM microstructure is negatively associated with antidepressant response (109), these clinical effects could also result from complex interactions between metabolism, WM integrity, and inflammatory markers.

## Immune Markers as Clinical Outcome and Diagnostic Predictors

The immune system is involved in BD, and the data described above seem to suggest a possible role in the etiology of the disorder, but there is more. Different studies reported how inflammatory markers can also help us to differentiate between mood disorders, illness phases, and predict treatment response. The first interesting data emerged from a meta-analysis that shows how peripheral concentrations of different cytokines remain elevated after antidepressant medication (e.g., escitalopram, venlafaxine, duloxetine) (110). Even if more than 50% of patients in the studies show clinical improvement after medication, cytokines modifications are very low. This evidence seems to underlie how cytokines can be strongly related to the disorder itself, and current medications only marginally affect their concentrations. Thus, altered levels of peripheral cytokines can probably be considered a stable biomarker of the disorder. BD patients show lower levels of plasma TNF-α and IL-13 (111), and serum IL-1β (112) compared to MDD patients. On the other hand, BD patients presented higher C-reactive protein, sTNFR1, soluble IL-6 receptor (sIL-6R), and MCP-1 levels compared to MDD patients (33, 113). Another group has tried to investigate the difference in the peripheral inflammatory markers' levels between BD illness phase. BD spectrum patients have higher TNF-α, TGF-β1, and IL-8 levels than controls, even if only IL-8 levels result significantly different in the BD spectrum, with higher levels in BD type I patients compared to BD type II and other unspecified BD (114). BD type I patients also show higher levels of the sTNF-R1 compared to BD type II patients (115). Although studies on the inflammatory differences between mood disorders and BD illness phase are currently too few to reach any conclusion, we cannot fail to notice that a difference in the inflammatory profile seems to exist.

More clinically relevant are some data on significantly different inflammatory profiles based on treatment response. In MDD patients, poorer medication response seems to be related to higher levels of circulating TNF-α (116), IL-6 (117, 118), IL-1 receptor (119), and acute-phase proteins (120). As previously suggested, these effects could both be due to the ability of cytokines to sabotage and circumvent mechanisms of action of conventional antidepressant agents on neurotransmitter function (121), and to their detrimental effects on synaptic plasticity and WM structure.

Concerning BD, one of the first predictive studies was performed by our group (122), showing a poorer antidepressant response to total sleep deprivation (TSD) treatment in those patients with higher baseline IL-6 serum levels. More recently, we investigated the predictive effect of a larger panel of cytokines, thus showing higher levels of proinflammatory IL-6, IL-8, IFN-γ, TNF-α, and MCP- 1 in TSD nonresponder patients compared to responders. Moreover, using individual component scores extracted from a principal component analysis performed on the five significant cytokines, we still find a negative effect of the factor scores on response to treatment (108).These data seem to support the key role of inflammatory factors, specifically proinflammatory cytokines, as biomarkers capable to differentiate between responder and not responder patients.

As further advances are made in this direction, we can hope to become able to identify a specific pattern of inflammatory alterations that allow early identification of treatment-resistant patients, to provide an effective tailored treatment as soon as possible.

## Conclusions

Recent works have thoroughly supported the association between BD and a proinflammatory state which involves both the innate and the adaptive immune system. In particular, T helper-1 cells (Th1), known to mediate cellular immune reactions, which induce production of cytokines such as IL-1, IL- 2, IL-6, TNF-α and IFN-γ, and Th2 cells, and enhance antibody-mediated reactions and the production of IL-4, IL-5, and IL-10, are heavily implicated. These results strongly suggest the existence of an inflammatory profile in BD patients during all phases of the disease. Indeed, compared to control subjects, BD patients show an increase in serum concentration of interleukins and CRP.

In a more specific way, Drexhage and colleagues demonstrated that BD patients and offspring share an inflammatory-genes mRNA overexpression signature that is detectable before illness onset (17, 18). Moreover, they state that inflammatory alterations occur not only in the periphery but also in the CNS, as demonstrated by microglial activation found both *in vivo* and postmortem in brains of BD patients (13, 14). This inflammatory condition can trigger a cascade of negative effects on brain structure and biology. In BD patients, cytokines levels and T cells percentage has been linked to WM structure alterations (55–57). These widespread alterations can also be linked to inflammatory-related oligodendrocyte dysfunction (46–49). WM modification has been related also to the tryptophan degradation cascade, that seems to be involved into the negative effect of an inflammatory condition (83). The increased levels of cytokines reduce the tryptophan-derived serotonin, increasing the production of tryptophan catabolites, which negatively impact on the neuroprotective ratio. These imbalances have been linked to an ineffective counter-regulating effect on the proinflammatory condition, and neurotoxicity (7, 78, 79). All these data seem to suggest that the immunological alteration concerning microglial, cytokines, and T cells, can represent a common etiological marker that partially explains the majority of the different modification founded as characteristics of BD.

Recently, another possible explanation has been explored in the relationship between the inflammatory profile and the hypothalamic-pituitary-adrenal axis (HPA) axis. Cytokines are capable of crossing the BBB and to act on various CNS areas, including this axis (7). HPA axis dysfunctions, together with elevated levels of proinflammatory cytokines can influence neuronal plasticity with a negative impact on mood symptoms and cognition. Moreover, higher levels of inflammatory cytokines may act by stimulating the HPA axis as a physiological response to stress (9). Taken together, this evidence goes on to highlight once again the notion that stress and synaptic plasticity might somehow be involved in the pathogenesis of mood disturbances, and especially BD.

Perhaps more interestingly, the role of inflammation in BD pathophysiology offers a new therapeutic approach, as targeting a reduction in the proinflammatory state of these patients might prove fruitful. Indeed, some research conducted with antiinflammatory drugs on other mood disorders, particularly MDD, has already shown a moderate degree of success (123–125), and their application in BD is attractive. Recent studies show that the characterization of peripheral inflammatory markers can lead to discriminate between responding and not responding to treatment patients, with the augmented levels of peripheral proinflammatory cytokines marks poorer antidepressant response (108, 122). We can, therefore, speculate that an early inflammatory characterization of BD patients, in particular of those patients that show higher immunological imbalance, can help to address a more tailored treatment to improve therapeutic efficacy. Further research is still needed to better understand the pathophysiological relation between inflammation and BD, to find new therapeutic approaches. Ongoing and future clinical studies with antiinflammatory agents will enlighten us on their therapeutic margin and help provide more arrows in our quiver against BD.

Regarding biological findings on peripheral markers, the vast difference and inconsistency among techniques used for analysis must be taken into consideration. If cytometry immunoprofilation does provide a wider idea on the main immune system features, showing with a high degree of precision which cell type is releasing particular cytokines and chemokines, a simple quantification like those performed using commercially available Elisa or panels won't be able to provide a clear and dependable overview of a patient's immune profile. Moreover, such biological data needs to be analyzed in larger multifactorial studies, with as many biomarkers as possible, for us to be able to understand the relations between the different factors involved in BD etiology and maintenance.

## Author Contributions

All individuals included as authors of papers contributed substantially to the scientific process leading up to the writing of the review. FB and RF designed the paper structure. VA, MP, and GG wrote the first draft of the manuscript. FB and RF supervised and revised the final version of the manuscript. All authors take final responsibility for the decision to submit for publication.

## Conflict of Interest

The authors declare that the research was conducted in the absence of any commercial or financial relationships that could be construed as a potential conflict of interest.
