# Supplementary material for: Neuroinflammation in Bipolar Depression
Source: Front Psychiatry. 2020 Feb 26;11:71. doi: 10.3389/fpsyt.2020.00071 (PMC7054443; doi:10.3389/fpsyt.2020.00071)
Supplement: Supplementary file 1 [file Image_1.pdf]

IL-6	TNF-α	sTNFR1	sTNFR2	CRP	IL-2	INF-γ	IL-10	IL-4	CCL11	CCL24	CXCL10	CXCL8	REF
↑													[21]
↑													[22]
	↑												[19]
	↑												[22]
	↑												[24]
	=												[25]
	=												[26]
		↑											[27]
			=										[27]
				↑									[28]
				↑									[29]
				↑									[30]
				=									[31]
					=								[21]
					=								[32]
						↓							[33]
						↑							[34]
							=						[21]
							=						[32]
								↑					[22]
									↑				[37]
										↑			[37]
											↑		[37]
												↓	[37]

Supplementary Figure 1. Key cytokines and chemokines alterations in BD patients compared with healthy controls. ↑ - increased ↓ - decreased = - no significant difference found. REF column is for reference to papers in which those specific cytokines were examined.
